# Age-Specific Transmissibility Change of COVID-19 and Associations With Breathing Air Volume, Preexisting Immunity, and Government Response

**DOI:** 10.3389/fpubh.2022.850206

**Published:** 2022-03-15

**Authors:** Qifa Song, Chao Cao, Yi Xiang, Liemin Ruan, Guoqing Qian

**Affiliations:** ^1^Medical Data Center, Ningbo First Hospital, Ningbo University, Ningbo, China; ^2^Department of Respiratory and Critical Medicine, Ningbo First Hospital, Ningbo University, Ningbo, China; ^3^Ningbo Women and Children's Hospital, Ningbo, China; ^4^Department of Infectious Diseases, Ningbo First Hospital, Ningbo University, Ningbo, China

**Keywords:** coronavirus disease 2019 (COVID-19), host susceptibility, volume of breathed air, age-specific transmissibility, preexisting immunity

## Abstract

**Background:**

The comprehensive impacts of diverse breathing air volumes and preexisting immunity on the host susceptibility to and transmission of COVID-19 at various pandemic stages have not been investigated.

**Methods:**

We classified the US weekly COVID-19 data into 0–4, 5–11, 12–17, 18–64, and 65+ age groups and applied the odds ratio (OR) of incidence between one age group and the 18–64 age group to delineate the transmissibility change.

**Results:**

The changes of incidence ORs between May, 2020 and November, 2021 were 0.22–0.66 (0–4 years), 0.20–1.34 (5–11 years), 0.39–1.04 (12–17 years), and 0.82–0.73 (65+ years). The changes could be explained by age-specific preexisting immunity including previous infection and vaccination, as well as volumes of breathing air. At the early pandemic, the ratio that 0–4-year children exhaled one-fifth of air and discharge a similar ratio of viruses was closely associated with incidence OR between two age groups. While, after a rollout of pandemic and vaccination, the much less increased preexisting immunity in children resulted in rapidly increased OR of incidence. The ARIMA model predicted the largest increase of relative transmissibility in 6 coming months in 5–11-year children.

**Conclusions:**

The volume of breathing air may be a notable factor contributing to the infectivity of COVID-19 among different age groups of patients. This factor and the varied preexisting greatly shape the transmission of COVID-19 at different periods of pandemic among different age groups of people.

## Introduction

As of November 2021, the severe acute respiratory syndrome coronavirus 2 (SARS-CoV-2) has caused more than 250 million cases of coronavirus disease 2019 (COVID-19) and 5.0 million deaths (1). The COVID-19 pandemic demonstrates distinctive age-specific infection rates (2), showing significantly lower infection rates in young children aged under 10 years at the early pandemic (3), as proved by the report of the first 149,082 US cases, consisting of only 2,572 (~1.7%) infants, children, and adolescents <18 years despite children <18 years making up 22% of the US population (4). This feature is contrasting with influenza, as reported that the odds ratio (OR) of influenza-like illness among children vs. adults was 3.1 (5), although both diseases are caused by respiratory RNA viruses alike.

Whether a virus can successfully invade individuals and causes symptoms depends on three determinant factors, including viral load reaching the host, environmental factors, and host susceptibility to the virus (6). Host susceptibility and viral load often demonstrate age-specific features (7). Previous studies have found similar distinctive age-specific infection rates of COVID-19 in many transmission scenarios (8, 9), suggesting that the distinctive host susceptibility between children and adults markedly accounts for lower transmissibility among children. Host susceptibility to infectious diseases usually depends on preexisting immunity and viral load entering individuals. Preexisting immunity to COVID-19 was determined by preceding infection and vaccination (10), both of which were more prominent in adults than in children because adults were prioritized for vaccination and had a higher incidence of COVID-19 in most countries.

As to the entrance of SARS-CoV-2 into the host, substantial evidence proved that airborne transmission through the respiratory route was the main mode of COVID-19 transmission (11). Airborne SARS-CoV-2 mainly comes from exhaled air of patients and previous studies have proved no difference in viral load in the breathing air between children and adults (12), hence, a higher volume of breathing air of patients results in a higher airborne viral load that greatly increases the transmissibility of COVID-19 (13). In human beings, the volume of breathing air is determined by breath rate and tidal volume. Tidal volume is the volume of air moving in or out of the lungs in one breathing cycle and is correlated with body weight, about 7 mL of breathing air per 1 kg body weight in one breath (14). A 70-kg adult breathes ~500 mL of air per breath or 10 L per min when he/she breathes 20 times per min at the rest state. Generally, children have a faster rate of breath, i.e., 30–40, 25–30, and 20–25 breaths/min for children under 1, 1–3, and 4–14 years, respectively. A child weighing 10 kg (1 year old, 30 breaths/min) approximately has a tidal volume of 70 mL and breathes 2.1 L of air per min (15).

To date, the comprehensive impacts of diverse breathing air volumes and preexisting immunity on the transmission of COVID-19 at different pandemic stages have not been investigated. Especially, the diverse breathing air volumes among different age people have been overlooked in previous studies. We hypothesized that at the early stages of the COVID-19 pandemic, when the whole population have very low immunity to SARS-CoV-2, the difference in breathing air volume was a determinant factor that characterizes the relative transmissibility of COVID-19 in terms of ORs of incidence between children and adults. While after a duration of pandemic and vaccination, the increased preexisting immunity would discretely affect the transmission in different age groups.

In this study, we used the age-stratified incidence of the US COVID-19 data to describe the change of relative host susceptibility and transmissibility among different age groups. Risk factors including different volumes of breathing air, preexisting immunity, and government responses were analyzed for host susceptibility and transmissibility during various stages of the pandemic. We also made a prediction of relative transmissibility among different age groups by the autoregressive integrated moving average (ARIMA) model.

## Methods

### Study Data

The COVID-19 weekly incidence of cases (number of cases/100,000 persons) and full vaccination rate (percentage) stratified by age were obtained from the US CDC (https://covid.cdc.gov/covid-data-tracker/#demographicsovertime). The data covered from May, 2020 to November, 2021. OR of incidence between an age group and 18–64 years group was calculated. Mean body weights of 0–4, 5–11, and 12–17 years were designated as 12, 25, and 48 kg that were the median body weight using the WHO children's standard weight. People of 18–64 and 65+ years were all adults and were designated a mean body weight of 70 kg. Their tidal volumes were calculated according to the equation of 7 mL × kg of body weight. The volume of breathing air per minute was calculated by tidal volume × breathing rate (Table 1). Ratio of volume of breathing air per minute between an age group and 18–64 years was calculated. Preexisting immunity in each age group was represented by the sum of infection percentage and full vaccination percentage.

**Table 1:** Body weight and volume of breathing air in different age individuals.

**Age group (year)**	**Weight (kg)**	**Tidal volume (mL)**	**Breathing rate (times/m)**	**Exhaled air/m (mL)**	**Ratio of volume of breathing air** ^**a**^
0– 4	12	84	30	2,520	0·26
5–11	25	175	25	4,375	0·45
12–17	48	336	20	6,720	0·69
18–64	70	490	20	9,800	1
65–	70	490	20	9,800	1

a *Ratio of volume of breathing air is calculated by comparing volume of exhaled air per min of an age group to that of the 18–64-year group*.

### Association Between Volume of Breathing Air and Incidence Among Different Age Populations

As airborne transmission through the respiratory route is the main mode of COVID-19 transmission, the transmissibility of COVID-19 is correlated with the viral load inhaled from the contaminated air. This viral load largely depends on the volume of exhaled or inhaled air by the patient. When children and adults were exposed to the same environment, such as in the household, they inhaled and exhaled age-specific viral loads because of different volumes of breathing air.

We used the breathing air volume calculated from body weight to represent the relative viral load transmitted *via* breathing air, which was referred to as the relative transmissibility in terms of OR. We designated the transmissibility of 70-kg adults to be 1. The ORs representing transmissibility and relative host susceptibility between one age group and 18–64-year adults were calculated.

### ARIMA Models

Time series analysis aims to reveal reliable and meaningful statistics and use this knowledge to predict future values of a series of data. The ARIMA model is one of the most used time series models and was applied in the analysis of COVID-19 transmission (16). The current study applied the automatic time series forecasting package in R to conduct ARIMA analysis (17). The weekly ORs between an age group and 18–64 age group were accompanied by time information and were typical time series data. We used these data to construct an ARIMA model to predict the change of OR in the following 26 weeks. The model that minimized the Akaike information criterion (AIC) amongst all appropriate models with different time period was selected as the best model for prediction.

## Results

**Figure 1:**
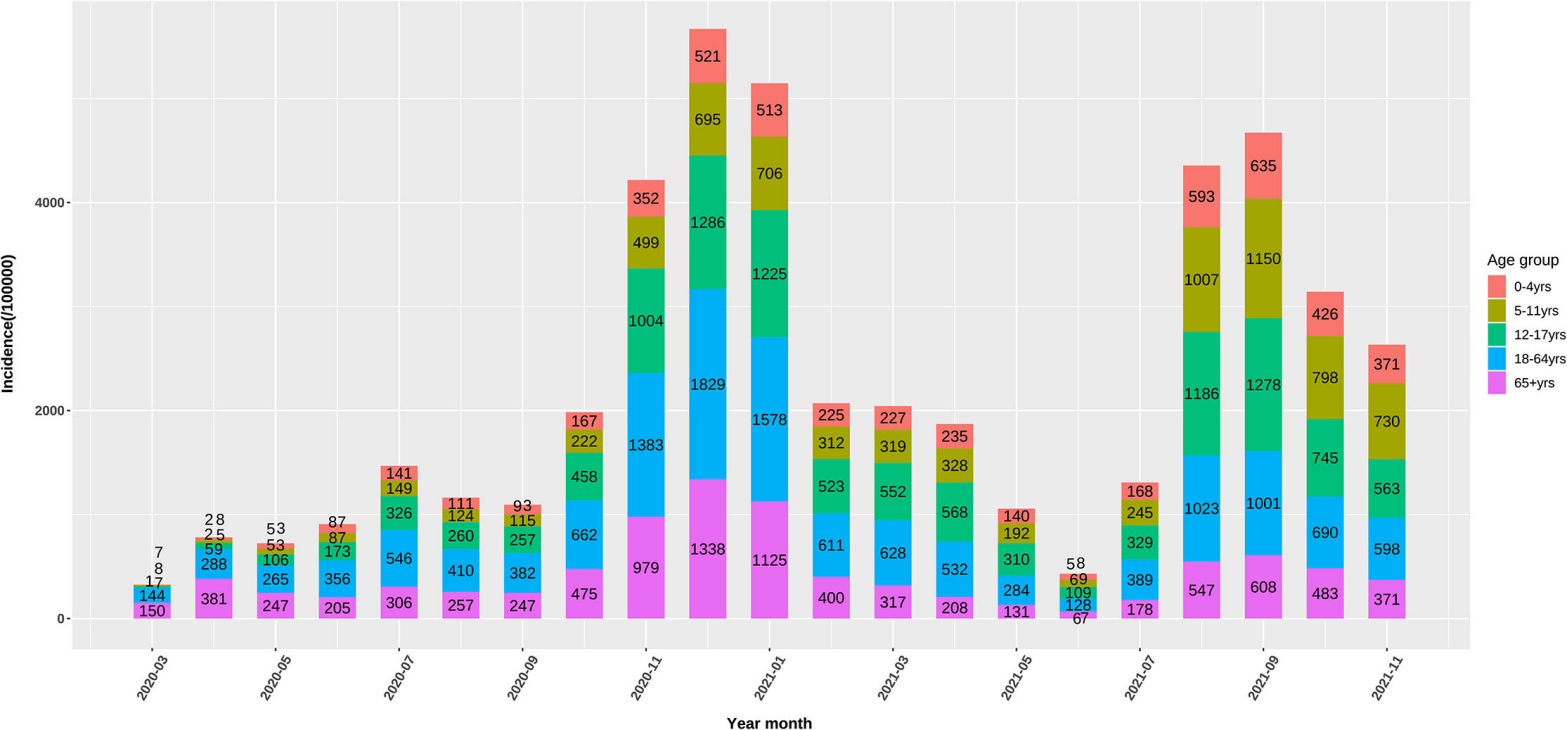
Monthly incidence (/100000) of the US COVID-19 confirmed cases in 0–4, 5–11, 12–17, and 65+ age groups.

**Figure 2:**
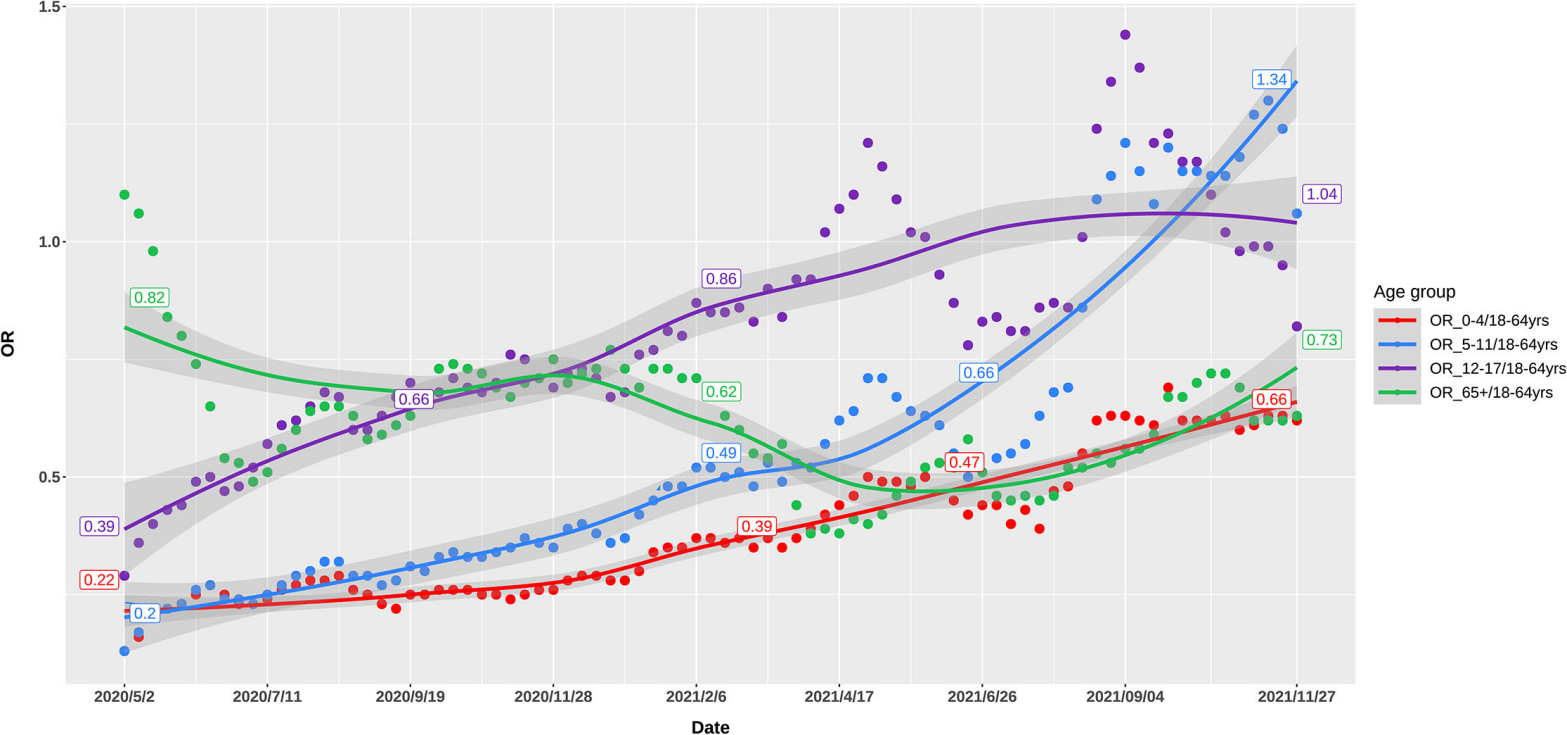
Odds ratio of weekly incidence of the US COVID-19 cases in 0–4, 5–11, 12–17, and 65+ age groups, as compared with 18–64 age group.

Up to November, 2021, 49 million cases were reported by the US CDC. Based on body weight, similar exposure chances in households, schools, and workplaces, we defined five age groups of 0–4, 5–11, 12–17, 18–64, and 65+ years by averaging the incidence of the component age groups. In May, 2020, 0–11-year children had the lowest risk of infection, while as the pandemic developed, the infection rate in school children (5–17 years) gradually increased to the high infection rates in November, 2021. The 65+ elders and 0–4 young children had low risk in the late pandemic (Figure 1). Compared with the 18–64 age group, the ORs of weekly incidence for 0–4, 5–11, and 12–17 age groups climbed from 0.22, 0.2, and 0.39 in May, 2020 to 0.66, 1.34, and 1.04 in November, 2021, respectively (Figure 2). This drastic raise of ORs indicated the relative increasing transmissibility of COVID-19 among the 0–17 years population. In contrast, the OR of the 65+ age group declined from 0.82 to 0.73 during the same period, suggesting a reduced relative susceptibility to COVID-19 in the 65+ years. Moreover, the 12–17 age group had two peaks of susceptibility increase around May and August, 2021.

**Figure 3:**
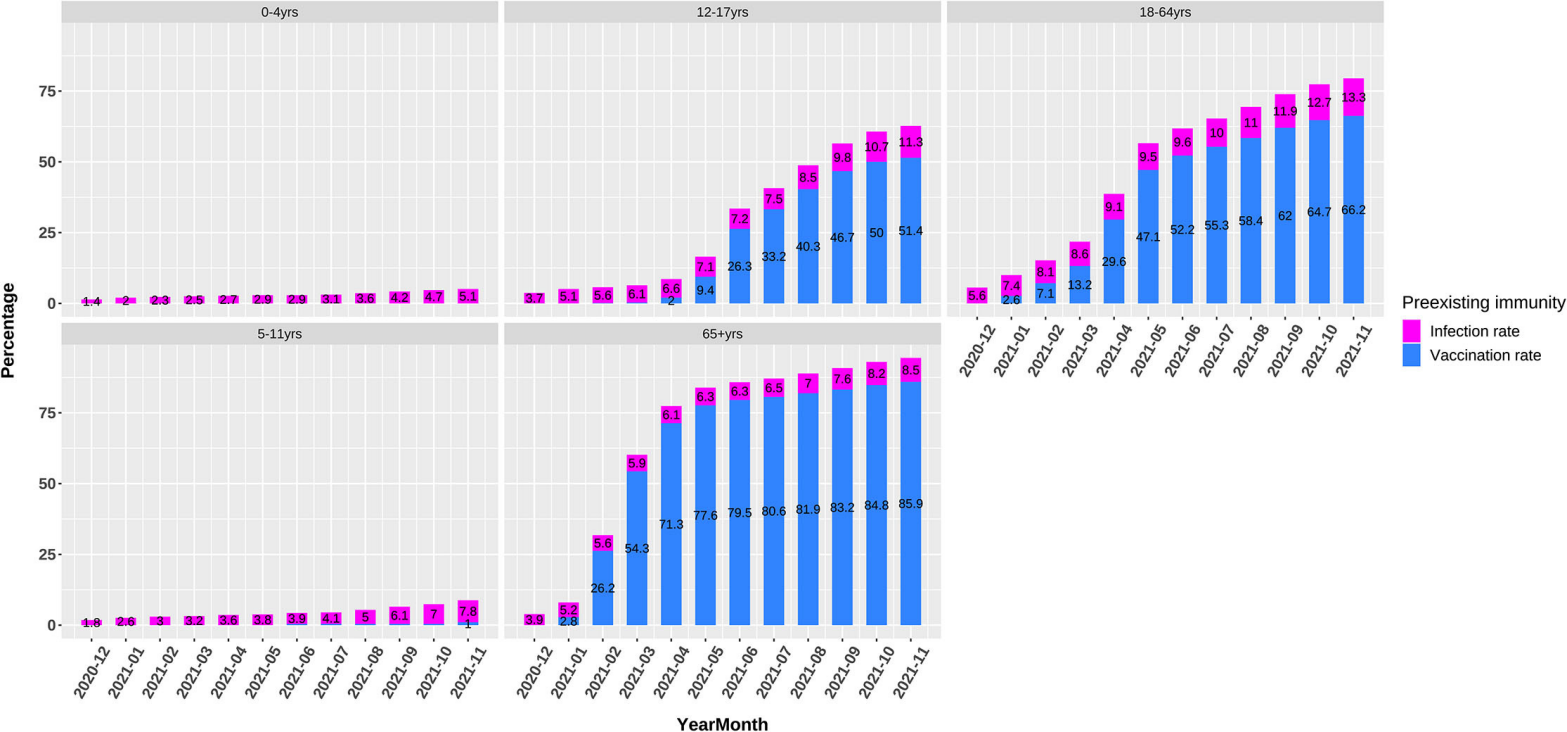
Preexisting immunity including infection and vaccination rates in 0–4, 5–11, 12–17, 18–64, and 65+ age groups.

In 2021, the vaccination was rolled out rapidly among the 12–17, 18–64, and 65+ age groups (Figure 3). As of November 2021, the full vaccination rates were 0, 1, 51.4, 66.2, and 85.9% among five age groups, respectively, indicating massively lower preexisting immunity rates among the 0–11-year children than the >12-year population.

As proposed above, when preexisting immunity has not been established at the early epidemic of an emerging respiratory virus like SARS-CoV-2, the airborne viral load inhaled and exhaled by patients was the key contributor to infection. Such a postulation was strongly proved by the evident relationship between OR of breathing air volume and OR of incidence at the early period of COVID-19 pandemic (Table 1). Compared with the volume of breathing air of 18–64-year adults, the ratios of breathing air volume for the 0–4, 5–11, 12–17, and 65+ age groups were 0.25, 0.45, 0.69, and 1, respectively. At the early pandemic period of May, 2020, the ORs of incidence of these age groups were 0.22, 0.20, 0.39, and 0.82, which was highly associated with the ORs of breathing air volume. For the 0–4-year children, the OR of incidence was very similar to the ratio of volume of breathing air, implicating the marked contribution of the volume of breathing air to host susceptibility. However, at the late pandemic period of November, 2021, the corresponding ORs of incidence in the 0–4, 5–11, 12–17 age groups were 0.66, 1.34, and 1.04. The increased OR of incidence in young children (0–4 and 5–11 years) suggested less contribution of breathing air volume to infection rate.

**Figure 4:**
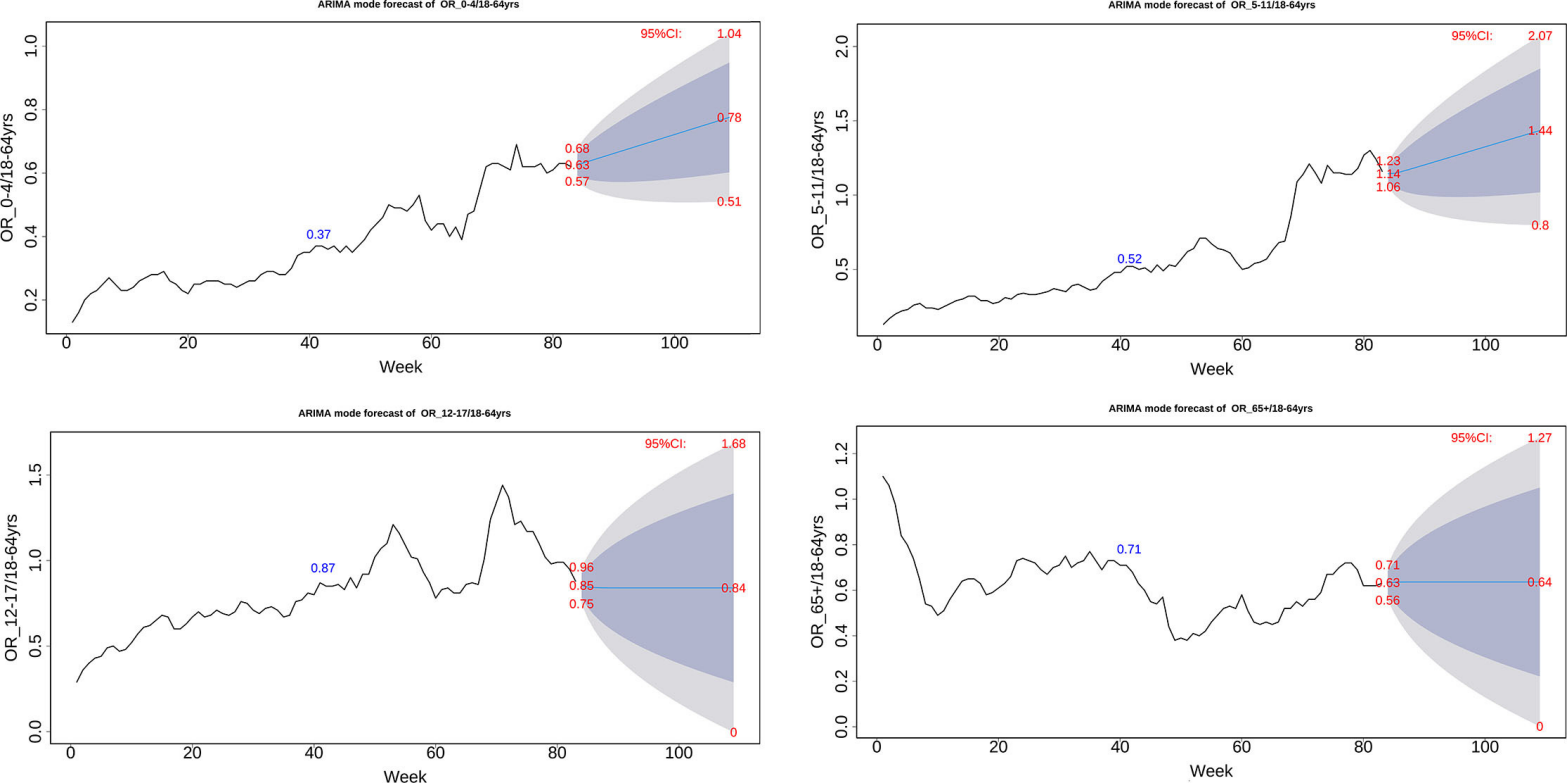
ARIMA model of weekly odds ratio of the US COVID-19 incidence between one age group and 64+ age group.

Finally, to investigate the OR trend denoting the relative host susceptibility of COVID-19 infection among different age groups, ARIMA model of weekly OR values of each age group was employed to make a 26-week prediction, i.e., from November, 2021 to May, 2022 (Figure 4). The model demonstrated that the 0–4 and 5–11 age groups would increase from 0.63 to 0.78, and from 1.14 to 1.4, respectively, while the 12–17 and 65+ age groups would remain a steady level. The results suggested that 0–11- year-young children would continuously experience an increased host susceptibility to COVID-19, an average increase by 26% as compared with adults.

## Discussion

We used the US weekly COVID-19 data to highlight the age-specific transmissibility change of COVID-19 resulting from host susceptibility during the COVID-19 pandemic. We demonstrated associations between host susceptibility and two risk factors of volume of breathing air and preexisting immunity. We conclude that at the early COVID-19 pandemic, varied volumes of breathing air are a notable positive determinant factor of host susceptibility and transmissibility of COVID-19, while at the late pandemic, preexisting immunity develops to be an increasing age-specific impact. The ARIMA model predicts a probable long-term increase of transmissibility in 0–11-year children.

By now, the age-specific transmissibility of SARS-CoV-2 requires further study. Previous research reported that children were less susceptible to COVID-19 infection than adults. Data from the first few months of the pandemic revealed that individuals aged under 20 years had approximately half of the infection rate of adults aged 20 years and older (18). However, these conclusions failed to describe the distinctive change of susceptibility among different age groups at different stages of the pandemic. This phenomenon also seemed puzzling when we considered that SARS-CoV-2 was RNA virus and had a similar respiratory transmission mode like influenza virus to which children are more susceptible. We undertook to figure out this discrepancy by investigating pivotal factors affecting host susceptibility. Besides the preexisting immunity, we included the volume of breathing air that varied dramatically between children and adults and determined the breathed viral load.

We applied OR of the incidence of COVID-19 between one age group and 18–64 age group to delineate the relative transmissibility among different age groups, as these OR values were more telling in demonstrating the changing incidence among various groups. These ORs changed differentially from the early to late COVID-19 pandemic, showing age-specific trajectories of host susceptibility change between age groups. As compared to 18–64 age group, the ORs of weekly incidence in 0–4, 5–11, and 12–17 age groups increased by 200, 570, and 266% from May, 2020 to November, 2021, respectively, while the OR for the 65+ age group declined by 11% (Figure 1). Notably, the 65+ age group had a rapid decrease in the OR around January–April, 2021, while 5–17 children had a rapid increase. Although mutated SARS-CoV-2 might partly account for the changes of transmissibility, the continuous age-specific OR changes implied persistently changing host susceptibility among different age groups.

As noted already, host susceptibility is chiefly affected by viruses successfully entering individuals and preexisting immunity. The magnitude of these two factors varied substantially among different age groups in a developing pandemic by an emerging pathogen. Our findings denoted that at the early stages of the COVID-19 pandemic when preexisting immunity was not established, the volume of breathing air played a pivotal role in determining the viral load exhaling from and inhaling into a patient and thus the transmissibility, as proved by the highly similar ORs between the volume of breathing air and the incidence among different age groups in May, 2020 (Table 1, Figure 2). The association between transmissibility and volume of breathing air could be easily inferred because the titration of the viruses in the air exhaled by adults and children was similar, while adults breathed more air (19). The ratio that a 70-kg adult breathes five times of air per minute as a 0–4-year child was almost the same as the OR of incidence between these two age groups. At the late stages of the pandemic, adults established a higher level of preexisting immunity due to higher infection rates and prioritized vaccination (Figure 3).

To what extent preexisting immunity can influence the transmissibility of COVID-19 among different age people for a long time remains a challenging question. We can get a clue from the characteristics of influenza transmission that rages on human beings for more than 100 years. Influenza infects people in many similar aspects such as transmission mode, mutation, as well as viral biological features. After years of fighting against influenza virus, adults are much less susceptible compared to children, owing to a higher-level preexisting immunity gradually established with age, as illustrated by a cross-sectional study in England in 2008 investigating baseline antibody titration to influenza A H1N1 infection. The England study showed age-dependent positive rates of baseline antibody as follows: 4.9% (0–4 years), 12.3% (5–14 years), 29.1% (15–24 years), 44.6% (50–64 years), and 83.7% (>80 years) (20), revealing great difference in baseline antibody levels among different age groups. This excellently explained the order of attack rate for 2009 pandemic influenza A (H1N1) that was 5–18, 0–4, 19–64, and 65+ years (21).

Considering the more than 1-year pandemic of COVID-19, preexisting immunity differed greatly between 18–64-year adults and 0–11-year children because of the age-specific vaccination policy and infection levels (Figure 3). We can view 0–4-year children as a baseline of preexisting immunity before a respiratory pandemic like SARS-CoV-2 and influenza virus because they do not experience enough exposure. When and how people have a profile of preexisting influenza-like immunity to COVID-19 is a vital issue to investigate and monitor. The ORs of different age groups in our study in November, 2021 showed an order trend partly like influenza, i.e., 5–11, 12–17, 18–64, 65+, and 0–4 age groups (Figures 1, 2). We found that the 65 + age group had preexisting immunity level of 94.4%, which was close to 2009 H1N1 (83.7%) in the preceding report. The current study suggests that preexisting immunity similar to influenza is developing in population in the COVID-19 pandemic.

Finally, ARIMA analysis made an age-specific prediction of the relative susceptibility. In the following 6 months, a steady increase in OR was predicted for the 0–4 and 5–11 age groups, whereas the 17–19 and 65+ age groups were predicted to remain an unchanged OR trend (Figure 4). We believe a longer time is required to reach transmission features like influenza that most age groups have a high level of preexisting immunity.

The present study had strengths and weak. The strength is that the American COVID-19 data have accurate information about age-specific incidence for the whole period. We used OR of incidence between age groups to vividly illustrate the relative transmissibility among different age groups. The weak aspect is that many confounding factors other than the three risk factors discussed above might affect the COVID-19 transmission. We managed to compensate other underlying factors using OR of incidence.

To summarize, our study highlights that varied preexisting immunity at the early and late stages of the COVID-19 pandemic wields distinctive impacts on people of different ages. The volume of breathing air may be a notable factor contributing to the infectivity of COVID-19 among different age groups of patients. Intensified monitor about the age-specific impacts of preexisting immunity on the transmissibility of the pandemic is imperative.

## Data Availability Statement

Publicly available datasets were analyzed in this study. This data can be found here: CDC (https://covid.cdc.gov/covid-data-tracker/#demographicsovertime).

## Ethics Statement

This methodological study was waived for ethical approval by the Ethics Committee of Ningbo First City Hospital as no patients were involved.

## Author Contributions

QS, CC, and YX designed the study and wrote the manuscript. LR and GQ provided the fund and reviewed the manuscript. QS analyzed the data and wrote the manuscript. All authors contributed to the article and approved the submitted version.

## Funding

This work was supported by Natural Science Foundation of Ningbo (2021J270), Ningbo City COVID-19 Epidemic Prevention and Control Project (202002N7033), and Public Welfare Fund of Zhejiang Province, China (No. LGF22G030010).

## Conflict of Interest

The authors declare that the research was conducted in the absence of any commercial or financial relationships that could be construed as a potential conflict of interest.

## Publisher's Note

All claims expressed in this article are solely those of the authors and do not necessarily represent those of their affiliated organizations, or those of the publisher, the editors and the reviewers. Any product that may be evaluated in this article, or claim that may be made by its manufacturer, is not guaranteed or endorsed by the publisher.
